# The application potential of *Gongronella* in agriculture: Status and challenges

**DOI:** 10.3897/imafungus.17.181825

**Published:** 2026-07-22

**Authors:** Junnan Fang, Li Li, Fei Li, Yuxin Zhu, Tingting Wang, Tingting Sun, Xiaoyong Liu, Xiaojie Wang, Zemin Fang

**Affiliations:** 1 School of Life Sciences and Medical Engineering, Anhui University, Hefei, Anhui 230601, China College of Life Sciences, Shandong Normal University Jinan China; 2 Anhui Key Laboratory of Biocatalysis and Modern Biomanufacturing, Hefei, Anhui 230601, China School of Life Sciences and Medical Engineering, Anhui University Hefei China; 3 College of Life Sciences, Shandong Normal University, Jinan 250358, China Anhui Key Laboratory of Biocatalysis and Modern Biomanufacturing Hefei China

**Keywords:** Global distribution, *
Mucorales
*, plant-fungus interactions, sustainable agriculture

## Abstract

*Gongronella*, a genus within the *Mucoromycota*, exhibits remarkable ecological adaptability and is widely distributed across diverse habitats. Recent research has revealed its capacity to colonize plant roots, stimulate plant growth, and enhance nutrient acquisition, underscoring its potential as both a biofertilizer and a biocontrol agent. *Gongronella* can secrete soil enzymes directly or recruit functional bacterial partners to cooperatively and efficiently decompose urea and organic phosphorus, thereby increasing nutrient availability and improving crop productivity. Its strong nutrient-mobilization capacity and resilience to environmental stresses position *Gongronella* as a promising microorganism for sustainable agriculture. Further investigation into its ecological functions and plant-associated interactions will advance our understanding of its symbiotic mechanisms and facilitate the development of environmentally friendly agricultural strategies.

## Introduction

Approximately 90% of terrestrial plants establish various symbiotic relationships with fungi in nature (Field et al. 2015; Martin et al. 2017). During these interactions, beneficial root-associated fungi can enhance mineral nutrient uptake and water-use efficiency, improve plant disease resistance, and promote plant growth (Behie and Bidochka 2014). In return, plants supply photosynthetic products to support fungal metabolism in the rhizosphere (Martin et al. 2017). Among the beneficial fungi associated with plants, the mycorrhizal and endophytic are two major guilds of symbionts that have received much attention (Wu et al. 2024; Zhen et al. 2024). Specifically, arbuscular mycorrhizal fungi (AMF) are the most widespread and ancient mutualists, having coexisted with plants for more than 400 million years. AMF are obligate biotrophs that significantly enhance plant nutrition, particularly phosphate uptake, whereas endophytic fungi colonize plant tissues without causing notable disease symptoms and can complete their life cycle independently of the host (Petrini 1991). Certain endophytic fungi, such as *Trichoderma* spp., also act as biocontrol agents, protecting plants from pathogens while promoting growth (Guzmán Guzmán et al. 2025). Fungal-plant symbioses play vital roles in improving plant nutrition, development, and stress tolerance, thereby enhancing agricultural productivity. These symbiotic benefits are critical for reducing chemical fertilizer and pesticide inputs, remediating heavy-metal-contaminated soils, controlling invasive species, and supporting food and environmental safety (Shamshiri and Fattahi 2016).

All AMF belong to *Glomeromycotina*, a subphylum under the phylum *Mucoromycota* (Spatafora et al. 2016). Another subphylum, *Mucoromycotina*, is also considered one of the earliest fungal lineages to establish symbiotic relationships with terrestrial plants (Bonfante and Venice 2020). This subphylum encompasses diverse ecological types, including saprotrophic, mycorrhizal, and pathogenic fungi, and it contains three major orders: *Endogonales*, *Mucorales*, and *Umbelopsidales* (Partida Martinez et al. 2007; Estrada-de Los Santos et al. 2018). The *Endogonales* have recently received considerable attention due to their unique ecological and physiological traits, which exhibit both ectomycorrhizal and arbuscular-like features, representing a key evolutionary link between early-diverging fungi and land plants (Fassi et al. 1969; Desirò et al. 2017; Hoysted et al. 2018). Fossil and molecular evidence confirm that *Mucoromycotina* fungi formed mutualistic associations with early land plants, providing essential nutrients such as phosphorus and nitrogen (Strullu Derrien et al. 2014; Field et al. 2016; Field and Pressel 2018).

Despite the growing recognition of fungal-plant interactions in agriculture, research on *Mucorales* remains limited. Within this lineage, the recently diverged genus *Gongronella* (23 million years ago) has attracted increasing interest due to its physiological versatility and ecological adaptability (Zhao et al. 2023; Wang et al. 2024a; Fang et al. 2025). Nevertheless, current knowledge of its functional mechanisms, symbiotic strategies, and potential applications remains fragmented. Addressing these gaps is essential to fully realize the agricultural value of Gongronella and integrate it into sustainable farming systems. This mini-review summarizes current knowledge on the morphological taxonomy of *Gongronella*, its distribution and phenotypic characteristics, plant growth-promoting activities, potential roles in bioremediation and pathogen suppression, and the challenges associated with its practical utilization.

## Taxonomic history and morphological characteristics of *Gongronella*

**Figure 1.**
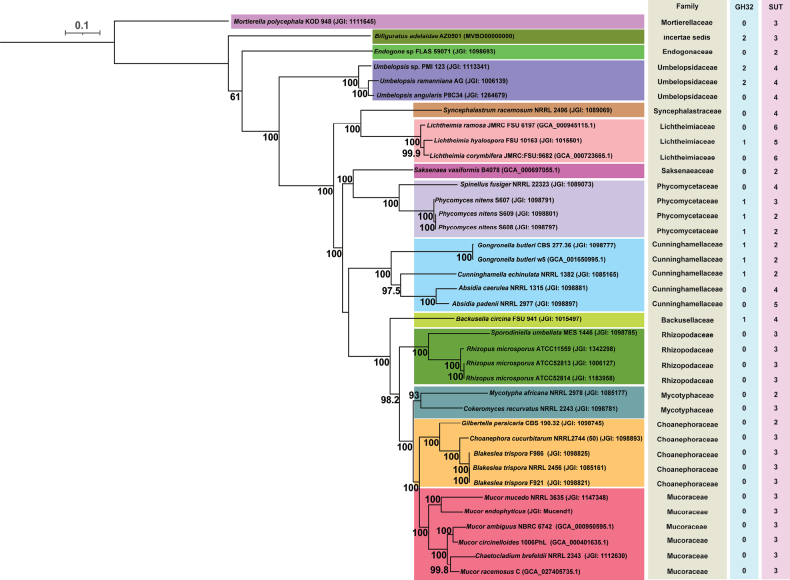
Phylogenomic relationships and functional gene distribution across *Mucoromycotina*. The phylogenomic tree was inferred from the concatenated amino acid sequences of 58 universal fungal marker genes using the Universal Fungal Core Genes (UFCG) pipeline and database (Kim et al. 2023). Taxon labels include the genus, species name, strain name, and their corresponding NCBI/JGI accession numbers. Currently, only two strains of *G. butleri* with publicly available genome sequences are included in the analysis. The numerical values at the nodes represent ultrafast bootstrap (UFBoot) support values from 1,000 replicates. The matrix on the right summarizes the presence and counts of key functional genes, specifically the GH32 (invertases) and SUT (sugar transporters) families. A “Family” column is provided to the right of the tree, and the background colors of taxon names correspond to these respective families. Note that *Bifiguratus adelaidae* AZ0501 currently lacks a formally assigned family but is placed within the order *Endogonales*.

The first member of the genus *Gongronella*, namely *G. butleri*, was first described by Lendner, who discovered this mysterious organism in the roots of *Cocos nucifera* on the Malay Peninsula and originally classified it under the genus *Absidia* Tiegh. 1878 as *A. butleri* Lendn. 1926. Ribaldi (1952) later successfully isolated the same fungus but described it as a new genus and species, *G. urceolifera*. Subsequently, Pici (1955) transferred *A. butleri* to *Gongronella*, giving it the current name *G. butleri* (Lend.) Peyronel & Dal Vesco, and treating *G. urceolifera* as its synonym (Hesseltine and Ellis 1964, 1961). Advances in molecular biology have further refined fungal systematics. Walther et al. (2013) conducted an in-depth phylogenetic analysis of *Absidia* and *Gongronella* using ITS and LSU rDNA sequence data, ultimately assigning both to the *Cunninghamellaceae* within the *Mucorales*, *Mucoromycotina* (Walther et al. 2013). To provide a robust evolutionary framework for this review, we reconstructed a phylogram of representative *Mucoromycotina* species using the Universal Fungal Core Genes (UFCG) pipeline (Kim et al. 2023). In brief, UFCG was used to identify and extract universal fungal core marker genes from genome assemblies. The extracted protein sequences were aligned, and columns with ≥50% gaps were removed. Of these, 58 shared marker genes from 38 genomes, comprising 34,917 amino acid positions, were retained and concatenated for phylogenetic inference. Maximum-likelihood phylogenomic analysis was performed using IQ-TREE v3.0.1. The best-fit amino acid substitution model was selected using ModelFinder according to the Bayesian Information Criterion (BIC), and the Q. YEAST+F+R6 model was identified as the optimal substitution model. Branch support was assessed using 1,000 ultrafast bootstrap (UFBoot) replicates and 1,000 SH-like approximate likelihood ratio test (SH-aLRT) replicates. *Mortierella polycephala* was utilized as a closely related outgroup to ensure precise rooting for *Mucoromycotina* (Fig. 1). Our analysis, which included two strains of *G. butleri* with available whole-genome sequences, further supported the close relationship of *Gongronella* with two members of *Absidia* (*A. caerulea* and *A. padenii*) in the *Cunninghamellaceae* (Ellis and Hesseltine 1965; Hesseltine and Ellis 1966) (Fig. 1).

Within this phylogenetic framework, we summarized the distribution of genes involved in sucrose metabolism in conjunction with available experimental evidence on carbon assimilation. As illustrated in Fig. 1, a distinct pattern of gene retention and loss emerged across lineages. Whereas sugar transporters (SUT) are ubiquitously present across the entire *Mucoromycotina* phylogeny, glycoside hydrolase family 32 (GH32, invertases) exhibit a heterogeneous distribution among various families. GH32 is predominantly retained in members of the *Cunninghamellaceae*, *Umbelopsidaceae*, *Lichtheimiaceae*, *Phycomycetaceae*, and *Backusellaceae*, while it is notably absent in the families *Mucoraceae*, *Chaetocladiaceae*, *Choanephoraceae*, *Mycotyphaceae*, *Rhizopodaceae*, and *Endogonaceae*. This observation aligns closely with the findings by Chang et al. (2022), who suggest that while GH32 genes were likely present in the common ancestor of zygomycetes, they underwent significant loss events across multiple lineages during diversification, whereas the transport machinery was broadly maintained.

However, experimentally validated data on carbon assimilation remain limited for most taxa included in this study. Among the available cases, *Gongronella butleri* w5 represents a well-characterized example in which the presence of both SUT and GH32 correlates with experimentally confirmed sucrose utilization capacity (Wang et al. 2024a; Fang et al. 2025). In contrast, most other taxa lack physiological characterization and therefore cannot be reliably annotated with carbon assimilation phenotypes. Within the *Mucoromycota*, including both *Mucoromycotina* and *Glomeromycotina*, experimental studies have shown that the absence of invertase activity (GH32) is associated with an inability to directly utilize sucrose, requiring host-mediated hydrolysis prior to monosaccharide uptake (Wipf et al. 2019). Given the limited availability of experimentally validated phenotypic data across taxa, these observations only indicate an association between GH32 presence and sucrose utilization capacity.

**Figure 2.**
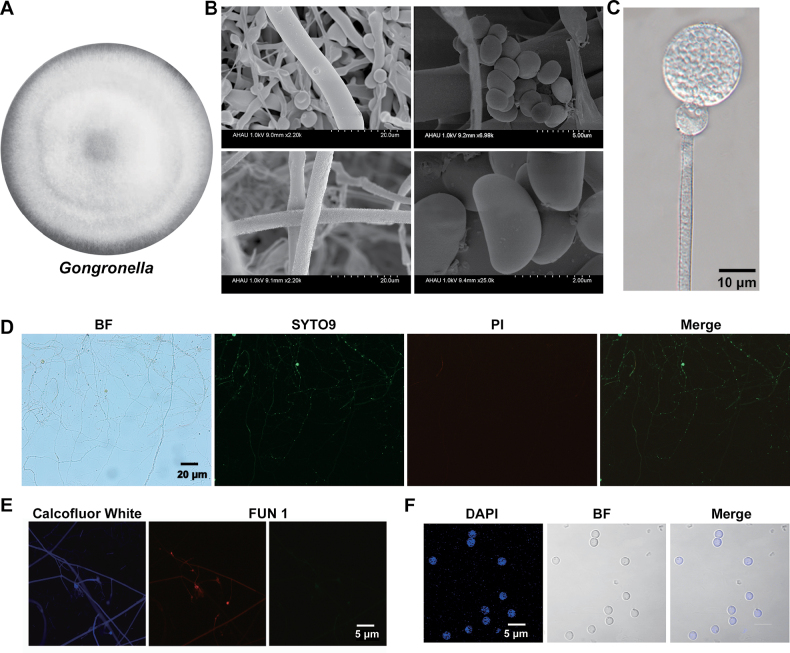
Colony morphology, microstructure, and endosymbiotic evidence of *Gongronella*. **A** Dense colony with smooth or lightly pigmented surfaces. **B** Scanning electron micrographs showing spherical to subspherical multispored sporangia and characteristic chlamydospores; the lower-left inset highlights the characteristic surface texture of mature vegetative hyphae. **C** Light microscopic photograph of a sporangium showing a characteristic apophasis (reproduced with permission, courtesy of Wang et al. (2024b)). **D** Intracellular occupancy of endofungal hyphal bacteria (EHB) within *G. butleri* w5. The presence of these symbionts was validated by dual-staining fluorescence microscopy. **E** Validation of fungal host viability and integrity. Hyphae were co-stained with FUN 1 and Calcofluor White M2R in an independent assay. Blue fluorescence (Calcofluor White) indicates intact cell walls; red fluorescence (FUN 1) represents high metabolic activity; and green fluorescence (FUN 1) denotes dead or inactive fungal cells. **F** Spore nuclei staining, DAPI (blue) marks the cell nuclei, while BF shows overall cell morphology.

Morphologically, *Gongronella* is characterized by a distinct swollen apophysis (Zhao et al. 2023). Hyphae and rhizoids are transparent to pigmented, highly branched, and grow slowly (Fig. 2B, C). Notably, certain strains, such as *G. butleri* w5, harbor a large number of endohyphal bacteria (EHB). The presence of bacterial-like signals associated with fungal hyphae was investigated using SYTO 9/propidium iodide (LIVE/DEAD BacLight kit) staining. In this assay, merged images of SYTO 9 (green) and PI (red) signals were used to visualize the presence of DNA-containing cells, including bacterial cells, associated with hyphae (Fig. 2D). To ensure signal accuracy and avoid spectral interference, fungal cell viability and hyphal structural integrity were assessed in parallel but with independent experiments using FUN 1 and Calcofluor White M2R staining, respectively (Fig. 2E). FUN1 staining showed red-orange intravacuolar fluorescence, consistent with metabolic conversion of the dye in viable fungal cells (Millard et al. 1997). The intracellular occupancy of EHB was interpreted based on fluorescence microscopy observations rather than ultrastructural confirmation, providing supporting evidence for bacterial association with fungal cells (unpublished data, communicated with Wang XJ).

Sporangiophores are produced from aerial hyphae, erect or slightly curved, unbranched or multiple branched, and occasionally contain a line of oil droplets. Sporangia are apophysate, spherical to subspherical, multispored, and smooth-walled. Chlamydospores vary in shape, spherical to ellipsoidal, and form laterally or at hyphal tips. Sporangiospores are multinucleate and vary in shape (Fig. 2F). Giant cells are intercalary in the rhizoids. Zygospores are brown to black, typically produced between two opposed and nearly equal suspensors (Hesseltine and Ellis 1961, 1964; Wang et al. 2023b; Wang et al. 2024b).

## Distribution and phenotypic characteristics

**Figure 3.**
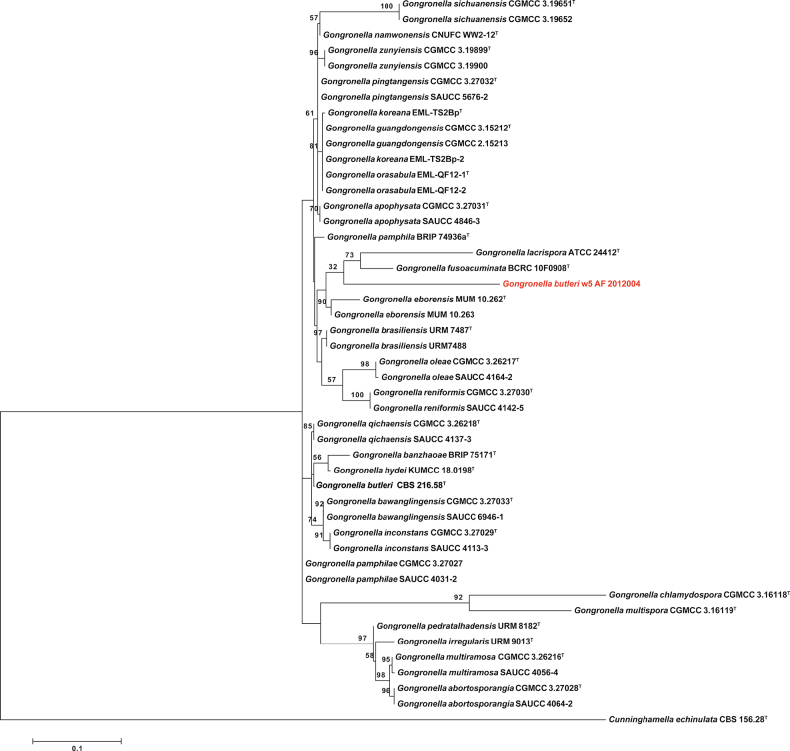
Phylogenetic tree of the genus *Gongronella*. The tree was constructed using the ML method with the Tamura-Nei model in MEGA 11. Bootstrap support values (1,000 replicates) are indicated at the nodes. *Cunninghamella echinulata* (CBS 156.28) served as the outgroup. All positions containing gaps and missing data were eliminated (complete deletion). The scale bar represents 0.1 substitutions per nucleotide position. Type and ex-type strains are indicated by the superscript “^T^” following the strain names. Strains shown in red indicate w5, which has been experimentally demonstrated to promote plant growth. The GenBank accession numbers for the ITS and LSU sequences used in this study are summarized in Suppl. material 2.

A complex interplay between potential global ubiquity and regional environmental filtering influences the distribution and diversity patterns of soil fungi. These patterns are shaped by various factors, including local ecological conditions, dispersal limitation, and evolutionary history (Tedersoo et al. 2014). A survey of taxonomic databases and recent literature reveals that the genus *Gongronella* is more diverse than previously reported. While Index Fungorum (https://www.indexfungorum.org/Names/Names.asp) and MycoBank (https://www.mycobank.org/Basic%20names%20search) list over 30 names, many represent nomenclatural synonyms or historical reclassifications. After a rigorous taxonomic audit, the genus is currently recognized to comprise approximately 30 accepted extant species. These include well-established taxa such as *G. butleri* and G*. lacrispora*, as well as numerous recently described species including *G. shangraoensis*, *G. yichunensis*, *G. qichaensis*, *G. abortosporangia*, and *G. banzhaoae*, among others (Table 1). To provide a historical perspective on this rapid taxonomic expansion, Table 1 organizes these species chronologically by their year of publication. Furthermore, based on the ITS and LSU sequences, a phylogenetic tree of the genus *Gongronella* was constructed. The phylogenetic position of *Gongronella* was determined based on a concatenated ITS and LSU dataset (approximately 1,465 bp). The Maximum Likelihood (ML) tree was reconstructed using MEGA 11 with the Tamura-Nei model and uniform rates among sites. The phylogeny was tested with 1,000 bootstrap replicates, and all positions containing gaps or missing data were excluded using the complete-deletion option. Heuristic search was conducted using the Nearest-Neighbor-Interchange (NNI) algorithm, with *Cunninghamella echinulata* (CBS 156.28) as the outgroup. The resulting ML tree (Fig. 3) revealed that all *Gongronella* species formed a monophyletic clade. Interestingly, strain w5 did not cluster with the type strain *G. butleri* CBS 216.58, but instead grouped within a subclade containing *G. fusoacuminata*BCRC 10F0908 and *G. lacrispora*ATCC 24412. Within this group, w5 showed closer phylogenetic proximity to *G. fusoacuminata*, although the corresponding bootstrap support was relatively low (32%), while the broader grouping with *G. lacrispora* was supported by a bootstrap value of 73%. This topological arrangement suggests a high degree of sequence conservation among these taxa and highlights the potential taxonomic complexity within the *G. butleri* species complex. Although several *Gongronella* isolates have been reported from plant-associated or disease-suppressive environments, experimental evidence supporting plant growth-promoting (PGP) activity remains extremely limited. To the best of our knowledge, strain w5 is currently the only *Gongronella* isolate whose PGP activity has been experimentally demonstrated. Consequently, the ecological occurrence of other *Gongronella* isolates should not be interpreted as evidence of plant growth-promoting function. The currently available data are therefore insufficient to determine whether PGP traits are broadly distributed across the genus or restricted to particular phylogenetic lineages. Future isolation and functional characterization of additional *Gongronella* strains will be essential for understanding the evolutionary distribution of plant-associated traits within this genus.

**Table 1.** Gongronella species recorded.

**Specie**s	**References**
*Gongronella butleri*	(Haynes et al. 1955)
*Gongronella lacrispora*	(Upadyay 1969)
*Gongronella koreana*	(Ariyawansa et al. 2015)
*Gongronella guangdongensis*	(Adamčík et al. 2015; Wang et al. 2023b)
*Gongronella orasabula*	(Li et al. 2016)
*Gongronella brasiliensis*	(Tibpromma et al. 2017)
*Gongronella butleri* w5	(Dong et al. 2018)
*Gongronella zunyiensis*	(Dong et al. 2019)
*Gongronella sichuanensis*	(Zhang et al. 2019b)
*Gongronella eborensis*	(Martins et al. 2020)
*Gongronella hydei*	(Doilom et al. 2020)
*Gongronella namwonensis*	(Crous et al. 2020)
*Gongronella pedratalhadensis*	(de Freitas et al. 2021)
*Gongronella banzhaoae*	(Wang et al. 2023b)
*Gongronella chlamydospora*	(Zhao et al. 2023)
*Gongronella multiramosa*	(Wang et al. 2023b)
*Gongronella multispora*	(Zhao et al. 2023)
*Gongronella oleae*	(Wang et al. 2023b)
*Gongronella qichaensis*	(Wang et al. 2023b)
*Gongronella pamphilae*	(Wang et al. 2024b)
*Gongronella abortosporangia*	(Wang et al. 2024b)
*Gongronella apophysata*	(Wang et al. 2024b)
*Gongronella bawanglingensis*	(Wang et al. 2024b; Tibpromma et al. 2017)
*Gongronella fusoacuminata*	(Wei et al. 2024; Wang et al. 2023b)
*Gongronella inconstans*	(Wang et al. 2024b; Adamčík et al. 2015)
*Gongronella irregularis*	(Crous et al. 2024)
*Gongronella pingtangensis*	(Wang et al. 2024b)
*Gongronella reniformis*	(Wang et al. 2024b)
*Gongronella yichunensis*	(Liu et al. 2025)
*Gongronella shangraoensis*	(Liu et al. 2025)

**Figure 4.**
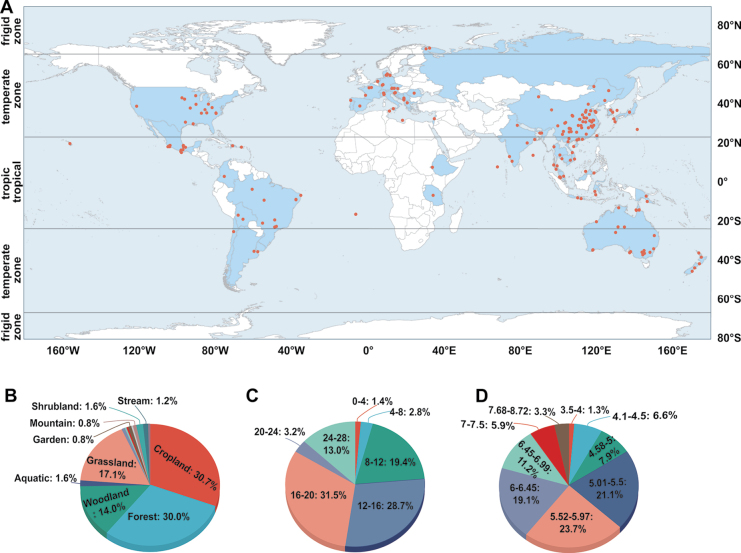
Global distribution and environmental preferences of *Gongronella*. **A** Global distribution of *Gongronella*. **B** Distribution of *Gongronella* among major ecosystem types, including forests, farmlands, grasslands, and woodlands. **C** Frequency of *Gongronella* occurrence across different mean annual temperature ranges. **D** Soil pH distribution. Detailed locality and environmental data are provided in Suppl. material 1.

The global distribution of *Gongronella* was mapped using a comprehensive dataset compiled from literature databases (including Web of Science, PubMed, CNKI, and Google Scholar) and the GlobalFungi database (https://globalfungi.com/). Using the R package (ggplot2, ggmap, sp, maptools, and maps) shows that the genus is widespread across tropical, subtropical, temperate, and cold regions, with primary habitats in subtropical and temperate zones, demonstrating its strong environmental adaptability (Fig. 4A). Biome analyses indicate that *Gongronella* is frequently isolated from forest, woodland, farmland, and grassland ecosystems, with occasional occurrences in shrub land or aquatic environments, suggesting a broad ecological range (Fig. 4B). In terms of temperature, *Gongronella* is most commonly found in regions with mean annual temperatures between 12 °C and 20 °C (Fig. 4C). It is often associated with slightly acidic soils (pH 5–6.5), suggesting a preference for, or tolerance of acidic conditions. In comparison, ectomycorrhizal fungi dominate in seasonally cold and dry climates, particularly at high latitudes and altitudes (Steidinger et al. 2019), and AMF are widely distributed in tropical forests, grasslands, and temperate forests (Öpik et al. 2006).

## *Gongronella* promoting plant growth

*Gongronella* has an excellent capacity for nutrient solubilization and activation. Studies have shown that *Gongronella* cooperate with mineral-dissolving bacteria such as *Bacillus thuringiensis* NL-11 to produce soil urease and phosphatase, which decompose urea and organic phosphorus, respectively, thereby releasing plant-available nutrients (Wang et al. 2023a). For example, *Gongronella* sp. JCS 16 solubilized nitrogen and phosphorus at concentrations of 10.48 ppm and 154.12 ppm, respectively (Mustika 2022). In addition, *Gongronella* secrete various organic acids that significantly increase the solubility of soil minerals, including calcium, magnesium, potassium, nitrogen, and phosphorus, thereby enriching the plant nutrient pool (Wu et al. 2017). Inoculation with *G. butleri* significantly increased soil urease, invertase, and peroxidase activities, leading to improvements in seedling height, stem diameter, leaf area, root length, and root volume in *Amorpha fruticosa* Lind (Wang et al. 2023a). Similarly, Jia et al. (2024) demonstrated that *G. butleri* enhanced mineral solubility, root nodulation, and growth in *Robinia pseudoacacia*. *Gongronella* has also been shown to promote growth and quality in chestnut plants (Chen et al. 2018). Furthermore, *G. butleri* w5 can penetrate kiwifruit root cells and secrete organic acids, facilitating phosphate acquisition and effectively promoting plant growth (Dong et al. 2018).

Notably, *Gongronella* associates with a wide range of host plants and can colonize rhizosphere soil or root tissues (Table 2). Since 1965, studies have discovered interactions between *Gongronella* and plants such as *Actinidia chinensis* (Fang et al. 2025), *Fallopia sachalinensis* (You et al. 2023), *Medicago sativa* (Wang et al. 2020), *Malus pumila* (Du et al. 2022), *Paeonia suffruticosa* (Mishra et al. 2016), *Quercus robur* (Santolamazza-Carbone et al. 2023), *Quercus virginiana* (Crockett 2023), *Salvia miltiorrhiza* (Li et al. 2023), sugarcane (Ren et al. 2024), *Gongronella* can also infect and colonize plant roots, serving as an endophyte in species such as *Cirsium arvense* (Kentjens et al. 2024), *Eucommia ulmoides* (Zhang et al. 2021), *Macadamia integrifolia* (Zakeel et al. 2023), and *Samanea saman* (Datu 2022). These findings highlight the ecological adaptability of *Gongronella* and suggest potential applications in agriculture.

**Table 2.** Overview of *Gongronella* species associated with different host plants, their colonization niches, and plant-growth-promoting effects.

**Plant host**	***Gongronella* species**	**Growth promotion**	**Colonizatin niche**	**Reference**
*Amorpha fruticosa*	*Gongronella butleri*	+	Rhizosphere	(Wang et al. 2023a)
*Robinia pseudoacacia*	*Gongronella butleri*	+	Rhizosphere	(Zhang et al. 2023)
*Actinidia chinensis*	*Gongronella* sp. w5	+	Rhizosphere	(Dong et al. 2018)
*Medicago truncatula*	*Gongronella* sp. w5	+	Rhizosphere	(Wang et al. 2020)
*Fallopia sachalinensis*	*Gongronella sichuanensis*		Rhizosphere	(You et al. 2023)
*Saccharum officinarum*	*Gongronella butleri*		Rhizosphere	(Ren et al. 2024)
*Quercus robur*	* Gongronella *		Rhizosphere	(Santolamazza-Carbone et al. 2023)
*Malus pumila*	* Gongronella *		Rhizosphere	(Du et al. 2022)
*Quercus virginiana*	*Gongronella* sp.		Rhizosphere	(Crockett 2023)
*Salvia miltiorrhiza*	* Gongronella *		Rhizosphere	(Sun et al. 2022)
*Melastoma malabathricum*	* Gongronella *		Root	(Mishra et al. 2016)
*Eucommia ulmoides*	* Gongronella *		Root	(Zhang et al. 2021)
*Macadamia integrifolia*	* Gongronella *		Root	(Zakeel et al. 2023)
*Samanea saman*	* Gongronella *		Root	(Datu 2022)
*Cirsium arvense*	* Gongronella *		Root	(Kentjens et al. 2024)
*Hevea brasiliensis*	*Gongronella butleri*		Rhizosphere	(Go et al. 2015)
*Aster sphathulifolius*	* Gongronella *		Root	(You et al. 2017)
*Carica papaya*	*Gongronella butleri*		Root	(Cruz-Lachica et al. 2018)
*Castanea mollissima*	*Gongronella butleri*	+	Root	(Chen et al. 2018)
*Gentiana scabra*	* Gongronella *		Rhizosphere	(Ge et al. 2014)
*Trifolium repens*	*Gongronella butleri*		Rhizosphere	(Thornton 1965)
*Lolium perenne*				
*Triticum aestivum*	*Gongronella butleri*		Rhizosphere	(Srivastava 1971)
*Hordeum vulgare*				
*Piper nigrum*	* Gongronella *		Root	(Watanabe et al. 1996)
*Syzygium malaccense*	*Gongronella* sp.		Root	(Hapida et al. 2021)
*Robinia pseudoacacia*	*Gongronella butleri*	+	Rhizosphere	(Li et al. 2021)
*Eucalyptus exserta*	*Gongronella* sp.		Root	(Mao et al. 2021)
*Eucommia ulmoides*	*Gongronella zunyiensis*		Rhizosphere	(Dong et al. 2019)
*Vaccinium* sp.	*Gongronella* sp. OP-2		Rhizosphere	(Hou et al. 2019)
	*Gongronella butleri*			
*Oryza sativa*	*Gongronella butleri*		Rhizosphere	(Seephueak et al. 2019)
*Phyllostachys heterocycla*	* Gongronella *		Rhizosphere	(Zhang et al. 2019a)

*Gongronella* establishes multifaceted associations with various plants through its extensive ecological adaptability. This genus exhibits a dual-niche colonization pattern, occupying both the rhizosphere and the endosphere. Once established, *Gongronella* contributes to mutualistic symbiosis by enhancing soil nutrient availability and supplying plants with essential minerals such as nitrogen and phosphorus. Such versatile interaction modes underscore the functional potential of *Gongronella* in promoting plant growth and its promise for sustainable agriculture.

Understanding the interaction dynamics and symbiotic mechanisms of *Gongronella* in host roots is crucial for its agricultural applications. In mycorrhizal symbioses, fungi activate and transport nutrients (Sun et al. 2022) and improve soil exploration by promoting root elongation and increasing root hair density (Nagy et al. 2005; Nagy et al. 2009; Teste et al. 2020). In return, plants provide carbohydrates to the fungi (Walder et al. 2012). Notably, mycorrhizal fungi cannot directly utilize sucrose and depend on plant cell wall invertases (CWInvs) to cleave sucrose into glucose and fructose for fungal cell growth (Ruan 2014; Roth and Paszkowski 2017; Wipf et al. 2019). Wang et al. (2020) reported a novel interaction mechanism in which *G. butleri* w5 directly assimilates and utilizes host-derived sucrose, offering a new perspective on fungal-plant interactions. Recent work further revealed that the sucrose transporter GspSUT1 and intracellular invertase GspINV from *G. butleri* w5 mediate sucrose uptake and allocation from plant roots, enabling the fungus to regulate carbon flow and shape the rhizosphere microbiome by enriching beneficial phosphate-solubilizing and nitrogen-fixing bacteria (Wang et al. 2024a; Fang et al. 2025). These findings highlight the central role of *Gongronella* in carbon metabolism and plant-microbe interactions, although the generality of this mechanism across the genus remains to be investigated.

## Potential roles of *Gongronella* in plant resistance against pathogenic microorganisms

*Gongronella* has been detected in diseased plant tissues or rhizospheres infected by various pathogens (Table 3). For instance, it has been found in *Morus alba* suffering from root rot (Gnanesh et al. 2021), *Annona muricata* affected by soursop rot (Zuraina et al. 2022), *Oryza sativa* with rice blast disease (Junaid et al. 2023), and *Solanum tuberosum* infected with nematodes (Zhang et al. 2023). While its presence in these symptomatic environments might initially suggest secondary colonization, additional ecological evidence suggests a functional role in plant health. Notably, recent studies indicate a significant negative correlation between *Gongronella* abundance and the incidence of fire blight disease in apple (*Malus pumila*) (Lee et al. 2023), black shank disease in tobacco (*Nicotiana tabacum*) (Wu et al. 2023), and black spot disease in edible roses (Dong et al. 2023), implying its potential role in enhancing soil suppressiveness against these pathogens. Beyond ecological associations, direct antagonism has been experimentally demonstrated against several major soil-borne fungi, including *Fusarium oxysporum* (Manjula et al. 2005), *Alternaria brassicicola* (cause of black spot in Chinese kale) (Komhorm et al. 2021), and *Fusarium solani* (cause of dry root rot in *Citrus*) (Chau et al. 2025). Apart from potentially suppressing pathogens, *Gongronella* shows potential for biocontrol of the root-knot nematode *Meloidogyne incognita* (dos Santos et al. 2022), the Asian corn borer *Ostrinia furnacalis* (Liu et al. 2025), and the beet armyworm *Spodoptera exigua* (Varela et al. 2017). These findings position *Gongronella* as a potential defensive component within the plant rhizosphere microbiome; however, its precise ecological roles and underlying anti-pathogenic mechanisms remain to be fully characterized.

**Table 3.** Reports of *Gongronella* associated with plant pathogen-infected tissues and diseased plants.

**Plant**	***Gongronella* species**	**Plant disease and pathogen**	**Reference**
*Annona muricata*	*Gongronella butleri*	soursop rot disease	(Zuraina et al. 2022)
*Oryza sativa*	*Gongronella* sp.	rice disease incidence	(Junaid et al. 2023)
*Solanum tuberosum*	* Gongronella *	nematode	(Zhang et al. 2023)
*Morus alba*	*Gongronella butleri*	root rot disease	(Gnanesh et al. 2021)
*Malus pumila*	*Gongronella butleri*	fire blight disease	(Lee et al. 2023)
*Nicotiana tabacum*	* Gongronella *	tobacco black shank	(Wu et al. 2023)
*Rosa rugosa*	* Gongronella *	black spot disease	(Dong et al. 2023)
*Beta vulgaris*	* Gongronella *	*Spodoptera exigua*	(Varela et al. 2017)
*Vitis vinifera*	*Gongronella butleri*	grapevine trunk diseases	(Bruez et al. 2016)
/	*Gongronella butleri*	*Meloidogyne exigua*	(Tazi et al. 2021)
*Elaeis guineensis*	*Gongronella* sp.	stem rot disease caused by *Ganoderma* spp.	(Kurniawan 2017)
*Fragaria ananassa*	*Gongronella* sp.	root rot disease	(Yuan et al. 2021)
*Morus alba*	*Gongronella butleri*	root rot (*Rhizopus oryzae*)	(Gnanesh et al. 2021)
Brassica oleracea var. acephala	*Gongronella butleri*	black spot disease *Alternaria brassicicola*	(Komhorm et al. 2021)
*Citrus nobilis*	*Gongronella butleri*	dry root rot	(Chau et al. 2025)

## Enzymatic resources of *Gongronella* and their potential in biotechnology and environmental remediation

The biotechnological value of *Gongronella* stems largely from its specialized cell wall biochemistry. Compared with traditional crustacean-derived chitosan, *Gongronella* mycelia provide a sustainable supply of chitosan with stable molecular characteristics, lower molecular weight, and freedom from shrimp allergens, making it highly suitable for pharmaceutical applications (Arcidiacono and Kaplan 1992; Ikeda et al. 1993; Tan 1996; Nwe and Stevens 2002; Nwe et al. 2009). In agriculture, these fungal-derived polymers and their oligosaccharide derivatives serve as potent biotic elicitors that can trigger plant innate immunity and systemic acquired resistance (SAR) against pathogens, while also stimulating root development and nutrient acquisition efficiency (Thambiliyagodage et al. 2023).

In addition to chitosan, *Gongronella* synthesizes a variety of enzymes, including chitin deacetylase, amylase, β-glucosidase, urease, carboxylase, and dehydrogenase, demonstrating its significant potential in biodegradation and organic waste treatment (Zhou et al. 2008; Wang et al. 2008; Fang et al. 2014; Santos et al. 2016; Cavalheiro et al. 2017; Valsalan et al. 2022). Temporiti et al. (2022) observed a significant increase in the relative abundance of *G. butleri* in plastic-contaminated environments, indicating its great potential for bio-composting. Similarly, *Gongronella* sp. WICC F60 can partially degrade low-density polyethylene, further suggesting its potential for plastic biodegradation (Dailin et al. 2024). Valsalan et al. (2022) reported that *G. butleri* isolated from organic compost produced higher levels of laccase and amylase than *Bacillus subtilis*, a common biosolid waste degrader. Martins et al. (2017) demonstrated that *Gongronella* can utilize formaldehyde as a carbon and energy source, effectively degrading it in soil. G*. butleri* also tolerates atrazine at concentrations up to 20 mg/L, and its high tolerance to metalaxyl may be linked to its strong degradation ability, particularly for methyl-group-containing compounds (Hock et al. 2020). A Vietnamese strain of *G. butleri* degrades polycyclic aromatic hydrocarbons (PAHs), which share structural similarities (ring systems) with atrazine, suggesting a general capacity for degrading aromatic pollutants (Ren et al. 2024). Mandl et al. (2018) found *Gongronella* exclusively in vineyard soils treated with glufosinate-ammonium, indicating potential for remediating herbicide-affected soils. *Gongronella* has been identified as a dominant, heavy-metal-tolerant genus across various extreme environments. In the Usangoda serpentine site (Sri Lanka), *G. butleri* demonstrated high experimental tolerance to Ni (up to 2,000 µg/mL) and Cu (up to 800 µg/mL), likely due to its production of chitosan, a natural metal chelator (Kumari and Saputhanthri 2019). Similarly, in the Russian Arctic, *G. butleri* was found to be the predominant fungal species in the air of industrial regions heavily contaminated by copper-nickel smelter emissions (Korneykova and Evdokimova 2018). These findings collectively provide strong ecological and experimental evidence for the genus’s robust tolerance to Cu and Ni, highlighting its potential for bioremediation in multi-metal polluted environments (Korneykova and Evdokimova 2018).

## Future perspectives on *Gongronella* utilization: Challenges and opportunities

*Gongronella* has attracted increasing attention due to its remarkable ability to promote plant growth and enhance nutrient acquisition. Compared to other well-studied beneficial fungi, such as arbuscular mycorrhizal fungi (AMF) and fine root endophytes (FRE), *Gongronella* possesses distinct biological attributes that enhance its agricultural potential. For example, AMF and many FRE are obligate biotrophs that require living host roots for propagation, leading to high production costs. By comparison, *Gongronella* is facultative, enabling cost-effective mass production through submerged fermentation (Wipf et al. 2019; Fang et al. 2025). Moreover, genomic data (Fig. 1) reveal that *Gongronella* retains a unique sucrose-processing toolkit (GH32/SUT), granting it metabolic independence and ecological fitness in complex soil environments compared to the hexose-dependent AMF.

Fully exploiting its potential requires addressing several challenges. The molecular mechanisms underlying *Gongronella*-plant interactions remain incompletely understood, including fungal recognition of plant signals, establishment of stable symbiosis, and modulation of plant physiology. However, the multinucleate nature of *Gongronella* spores poses a significant hurdle for molecular genetic manipulation, as it often leads to heterokaryon formation during transformation, making the isolation of stable, homokaryotic mutant lines extremely difficult. Furthermore, the complex microbial associations, including putative endosymbiotic bacteria typical of *Mucoromycota* (Bonfante and Desirò 2017), add another layer of complexity that precludes the use of conventional resistance-marker-based genetic tools. It should be noted that recent studies have successfully applied host-induced gene silencing (HIGS) to investigate *Gongronella* functions (Fang et al. 2025), providing a promising avenue for dissecting its molecular mechanisms.

The genomic and multi-omic landscape of *Gongronella* remains under-explored. While the genome of *G. butleri* w5 has provided preliminary insights into its metabolic repertoire, particularly regarding carbohydrate-active enzymes (CAZymes) and potential plant-growth-promoting traits (Dong et al. 2018), much of its functional potential remains to be fully elucidated. As highlighted by Almario et al. (2022), multi-omics approaches, including genomics, transcriptomics, and metabolomics, are indispensable for deciphering the nutrient “quid pro quo” and the molecular dialogue between roots and beneficial fungi. Future research should prioritize comparative genomics with related mucoralean taxa to identify unique genomic signatures associated with nutrient mobilization and environmental resilience. Integrating these high-throughput approaches with experimental validation will be key to identifying the core gene clusters responsible for its multifaceted ecological functions.

Beyond direct nutrient activation, *Gongronella* dynamically modulates the rhizosphere microbiome by recruiting functional bacteria (Wang et al. 2024a; Fang et al. 2025). *G. butleri* w5 employs a specific sucrose transporter to uptake plant-derived sucrose, which is subsequently hydrolyzed to release fructose into the rhizosphere. Fructose then serves as a specialized carbon source that selectively recruits and sustains beneficial microbes, particularly phosphate-solubilizing and nitrogen-fixing bacteria. By fostering these functional groups, *Gongronella* facilitates a synergistic supply of essential macronutrients (P and N), thereby significantly bolstering plant growth. This multi-layered interaction highlights its central role in root-associated ecosystems and provides a conceptual framework for designing microbe-driven sustainable agricultural strategies. Understanding this tripartite interaction among *Gongronella*, soil microorganisms, and host plants is crucial for optimizing its application and ecological function.

In addition, the enzymatic diversity, bioactive metabolites, and environmental resilience of these fungi may confer substantial potential for bioremediation, including organic waste decomposition, plastic degradation, pesticide residue removal, and heavy metal detoxification. Fully realizing these applications requires a comprehensive understanding of their metabolic pathways, using multi-omics approaches (e.g., genomics, transcriptomics, and proteomics) to identify key functional genes and enzymes involved in pollutant breakdown. Furthermore, optimizing practical application methods (such as seed coating or fertigation) and understanding interactions with coexisting soil microbiota, complemented by large-scale field trials, are essential to assess practical efficacy (Fig. 4).

Future research should focus on: (i) elucidating molecular mechanisms of signal recognition, carbon allocation, and maintenance of symbiotic homeostasis with host plants; (ii) evaluating functional performance across diverse soils, crops, and environmental conditions, including strategies for recruiting and optimizing beneficial soil microbial communities; and (iii) conducting field-scale validations of its agricultural and remediation potential. Integrating molecular, ecological, and applied approaches will provide a solid scientific foundation for the safe, efficient, and sustainable utilization of *Gongronella*, expanding its prospects in green agriculture and environmental management. Building on these biological insights, it is essential to develop practical strategies to transition *Gongronella* from laboratory discovery to large-scale agricultural deployment.

## Practical strategies for agricultural deployment and field transition

While functional evidence for the genus has predominantly been derived from controlled pot experiments, primarily with crops like tobacco and tomato, large-scale field applications remain in their infancy. To bridge the gap between laboratory proof-of-concept and realistic agricultural deployment, we propose three primary scenarios based on the biological strengths and inter-domain synergies identified in strain w5. The first scenario involves the development of synergistic bio-organic fertilizers. Given its high cellulolytic activity and resilience in organic-rich environments, *Gongronella* is a preferred candidate for integration into compost-based or biochar-based fertilizers. This carrier-based approach protects the fungal mycelia from competition with indigenous soil microbes (Kaur and Kaur 2023), while simultaneously leveraging the fungus to accelerate organic matter turnover. The second strategy is the application of targeted seed coating, in which *Gongronella* spores are applied directly to seeds to ensure early colonization of the spermosphere. By enhancing nutrient availability during critical early growth stages, this method provides emerging seedlings with a localized metabolic advantage (Rocha et al. 2019). The third approach focuses on the construction of fungal-bacterial composite consortia. Recent research has elucidated the molecular basis for the cooperativity between *Gongronella* and the rhizosphere microbiome mediated by specific fungal sucrose transporters that coordinate the “carbon-for-nutrient” exchange among the plant, fungus, and bacteria. Leveraging these established cross-feeding mechanisms, the development of synthetic communities that pair up *Gongronella* with specific bacterial partners offers a robust strategy for field deployment.

**Figure 5.**
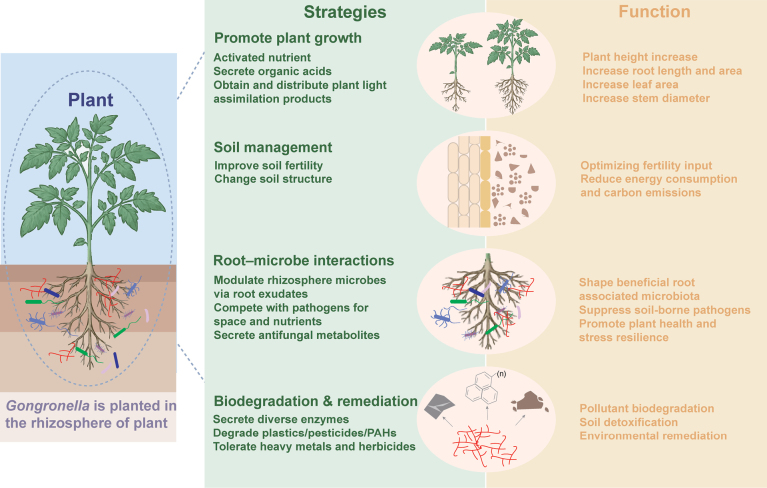
Multifaceted ecological and biotechnological functions of *Gongronella*. This schematic summarizes the diverse roles of *Gongronella* in nutrient mobilization, plant growth promotion, and environmental bioremediation. To facilitate the establishment of the fungus in the rhizosphere, several practical inoculation approaches can be employed, including seed coating with fungal spores, seedling root dipping, soil amendment using granular inoculants, and fertigation through irrigation systems. These methods ensure effective fungal-plant associations and the subsequent expression of their beneficial functions.

Transitioning to field efficacy will inevitably face challenges such as environmental fluctuations and microbial antagonism. Future research should prioritize active field inoculation trials to determine the optimal dosage, timing, and soil-specific compatibility of these fungal-bacterial consortia, moving beyond mere taxonomic detection toward stable and reproducible agricultural performance (Fig. 5).

## Additional information

### Artificial Intelligence (AI) use

The authors accept full responsibility for the content of the manuscript, including the disclosure of any use of AI.

No AI tools were used in the preparation of this manuscript.

### Adherence to national and international regulations

All the fungal strains used in this study have been legally obtained, respecting the Convention on Biological Diversity (Rio Convention).

### Author ORCIDs

Junnan Fang https://orcid.org/0009-0000-4463-6885

Li Li https://orcid.org/0000-0002-8454-2952

X. Liu https://orcid.org/0000-0002-8808-010X

Xiaojie Wang https://orcid.org/0000-0002-8102-4658

Z. Fang https://orcid.org/0000-0002-9853-5470

### Data availability

This article is licensed under a Creative Commons Attribution 4.0 International License, which permits use, sharing, adaptation, distribution and reproduction in any medium or format, as long as you give appropriate credit to the original author(s) and the source, provide a link to the Creative Commons licence, and indicate if changes were made. The images or other third party material in this article are included in the article’s Creative Commons licence, unless indicated otherwise in a credit line to the material. If material is not included in the article’s Creative Commons licence and your intended use is not permitted by statutory regulation or exceeds the permitted use, you will need to obtain permission directly from the copyright holder. To view a copy of this licence, visit http://creativecommons.org/licenses/by/4.0/.

## Supplementary materials

Supplementary material 1 Global distribution and environmental preferences of *Gongronella*: Detailed locality and ecological parameters Data type xlsx https://binary.pensoft.net/file/1723120This dataset is made available under the Open Database License (http://opendatacommons.org/licenses/odbl/1.0/). The Open Database License (ODbL) is a license agreement intended to allow users to freely share, modify, and use this Dataset while maintaining this same freedom for others, provided that the original source and author(s) are credited. Junnan Fang, Li Li, Fei Li, Yuxin Zhu, Tingting Wang, Tingting Sun, Xiaoyong Liu, Xiaojie Wang, Zemin Fang

Supplementary material 2 Additional information Data type xlsx https://binary.pensoft.net/file/1723121This dataset is made available under the Open Database License (http://opendatacommons.org/licenses/odbl/1.0/). The Open Database License (ODbL) is a license agreement intended to allow users to freely share, modify, and use this Dataset while maintaining this same freedom for others, provided that the original source and author(s) are credited. Junnan Fang, Li Li, Fei Li, Yuxin Zhu, Tingting Wang, Tingting Sun, Xiaoyong Liu, Xiaojie Wang, Zemin Fang

## References

[B1] Adamčík S, Cai L, Chakraborty D et al. (2015) Fungal biodiversity profiles 1–10. Cryptogamie, Mycologie 36(2): 121–166. 10.7872/crym/v36.iss2.2015.121

[B2] Almario J, Fabiańska I, Saridis G et al. (2022) Unearthing the plant-microbe quid pro quo in root associations with beneficial fungi. New Phytologist 234(6): 1967–1976. 10.1111/nph.18061 35239199

[B3] Arcidiacono S, Kaplan DL (1992) Molecular weight distribution of chitosan isolated from *Mucor rouxii* under different culture and processing conditions. Biotechnology and Bioengineering 39(3): 281–286. 10.1002/bit.260390305 18600943

[B4] Ariyawansa HA, Hyde KD, Jayasiri SC et al. (2015) Fungal diversity notes 111–252-taxonomic and phylogenetic contributions to fungal taxa. Fungal Diversity 75: 27–274. 10.1007/s13225-015-0346-5

[B5] Behie SW, Bidochka MJ (2014) Nutrient transfer in plant-fungal symbioses. Trends in Plant Science 19(11): 734–40. 10.1016/j.tplants.2014.06.007 25022353

[B6] Bonfante P, Desirò A (2017) Who lives in a fungus? The diversity, origins and functions of fungal endobacteria living in *Mucoromycota*. ISME Journal 11(8): 1727–1735. 10.1038/ismej.2017.21 PMC5520026 28387771

[B7] Bonfante P, Venice F (2020) *Mucoromycota*: going to the roots of plant-interacting fungi. Fungal Biology Reviews 34(2): 100–113. 10.1016/j.fbr.2019.12.003

[B8] Bruez E, Baumgartner K, Bastien S et al. (2016) Various fungal communities colonise the functional wood tissues of old grapevines externally free from grapevine trunk disease symptoms. Australian Journal of Grape and Wine Research 22(2): 288–295. 10.1111/ajgw.12209

[B9] Cavalheiro GF, Sanguine IS, Santos FRDS et al. (2017) Catalytic properties of amylolytic enzymes produced by *Gongronella butleri* using agroindustrial residues on solid-state fermentation. BioMed Research International 2017: 7507523. 10.1155/2017/7507523 PMC5742443 29376074

[B10] Chang Y, Wang Y, Mondo S et al. (2022) Evolution of zygomycete secretomes and the origins of terrestrial fungal ecologies. iScience 25(8). 10.1016/j.isci.2022.104840 PMC9391592 35996588

[B11] Chau ATT, Nguyen NT, Tat AT et al. (2025) Efficacy of bio-composted agricultural wastes and biocontrol agents in improving soil fertility and controlling dry root rot in King mandarin (*Citrus nobilis*) orchards. Agriculture and Natural Resources 59(4): 590403–590403. https://li01.tci-thaijo.org/index.php/anres/article/view/268615

[B12] Chen Y, Wu J, Xu B et al. (2018) Isolation and identification of ectomycorrhizal fungi from *Castanea mollissima*. Mycol Soc China.

[B13] Crockett H (2023) Molecular identification of oomycete species associated with woody plants in Louisiana and survey of oomycete species associated with live oak trees planted on the Louisiana State University campus. Louisiana State University and Agricultural & Mechanical College

[B14] Crous P, Wingfield M, Chooi YH et al. (2020) Fungal Planet description sheets: 1042–1111. Persoonia 44: 301–459. 10.3767/persoonia.2020.44.11 PMC7567971 33116344

[B15] Crous P, Wingfield M, Jurjević Ž et al. (2024) Fungal Planet description sheets: 1697–1780. Fungal Systematics and Evolution 14: 325–577. 10.3114/fuse.2024.14.19 PMC11736264 39830292

[B16] Cruz-Lachica I, Marquez-Zequera I, Allende-Molar R et al. (2018) Diversity of mucoralean fungi in soils of papaya (*Carica papaya* L.) producing regions in Mexico. Fungal Biology 122(8): 810–816. 10.1016/j.funbio.2018.04.008 30007431

[B17] Dailin DJ, Azzahra SZ, Rithwan F et al. (2024) Screening of different fungi strains *Gongronella* sp. WICC F60 and *Cordyceps* sp. WICC F61 for degradation of low-density polyethylene. Journal of Bioprocess and Biomanufacturing Technology 3(1): 21–25. 10.11113/bioprocessing.v3n1.46

[B18] Datu IT (2022) Identifikasi keragaman cendawan pada jaringan pohon trembesi (*Samanea saman*) di universitas Hasanuddin, Makassar. Universitas Hasanuddin

[B19] de Freitas LWS, de Oliveira RJV, Leite TRC et al. (2021) *Gongronella pedratalhadensis*, a new species of *Mucorales (Mucoromycota)* isolated from the Brazilian. Sydowia 73: 61.

[B20] Desirò A, Rimington WR, Jacob A et al. (2017) Multigene phylogeny of *Endogonales*, an early diverging lineage of fungi associated with plants. IMA Fungus 8(2): 245–257. 10.5598/imafungus.2017.08.02.03 PMC5729711 29242774

[B21] Doilom M, Guo JW, Phookamsak R et al. (2020) Screening of phosphate-solubilizing fungi from air and soil in Yunnan, China: four novel species in *Aspergillus*, *Gongronella*, *Penicillium*, and *Talaromyces*. Frontiers in Microbiology 11: 585215. 10.3389/fmicb.2020.585215 PMC7574596 33123114

[B22] Dong CB, Zhang ZY, Chen WH et al. (2019) *Gongronella zunyiensis* sp. nov.(*Cunninghamellaceae*, *Mucorales*) isolated from rhizosphere soil in China. Phytotaxa 425(5): 290–296. 10.11646/phytotaxa.425.5.4

[B23] Dong W, Long T, Ma J et al. (2023) Effects of *Bacillus velezensis* GUAL210 control on edible rose black spot disease and soil fungal community structure. Frontiers in Microbiology 14: 1199024. 10.3389/fmicb.2023.1199024 PMC10415101 37577414

[B24] Dong Y, Sun Q, Zhang Y et al. (2018) Complete genome of *Gongronella* sp. w5 provides insight into its relationship with plant. Journal of Biotechnology 286: 1–4. 10.1016/j.jbiotec.2018.08.022 30194967

[B25] Dos Santos AMM, De Souza LM, Da Silva JF et al. (2022) Potential of fungal isolates to reduce *Meloidogyne incognita* parasitism in tomato. Brazilian Journal of Development 8(2): 13942–13956. 10.34117/bjdv8n2-361

[B26] Du P, Cao Y, Yin B et al. (2022) Improved tolerance of apple plants to drought stress and nitrogen utilization by modulating the rhizosphere microbiome via melatonin and dopamine. Frontiers in Microbiology 13: 980327. 10.3389/fmicb.2022.980327 PMC9687389 36439851

[B27] Ellis J, Hesseltine C (1965) The genus *Absidia*: globose-spored species. Mycologia 57(2): 222–235. 10.1080/00275514.1965.12018205

[B28] Estrada-de los Santos P, Palmer M, Chávez-Ramírez B et al. (2018) Whole genome analyses suggests that *Burkholderia* sensu lato contains two additional novel genera (*Mycetohabitans* gen. nov., and *Trinickia* gen. nov.): implications for the evolution of diazotrophy and nodulation in the *Burkholderiaceae*. Genes 9(8). 10.3390/genes9080389 PMC6116057 30071618

[B29] Fang J, Wang X, Li L et al. (2025) A sucrose transporter from *Gongronella butleri* w5 mediates plant-fungus-bacteria interaction. Current Biology. 10.1016/j.cub.2025.08.043 40961929

[B30] Fang W, Song R, Zhang X et al. (2014) Characterization of a novel β-glucosidase from *Gongronella* sp. w5 and its application in the hydrolysis of soybean isoflavone glycosides. Journal of Agricultural and Food Chemistry 62(48): 11688–11695. 10.1021/jf502850z 25389558

[B31] Fassi B, Fontana A, Trappe JM (1969) Ectomycorrhizae formed by *Endogone lactiflua* with species of *Pinus* and *Pseudotsuga*. Mycologia 61(2): 412–414. 10.1080/00275514.1969.12018748

[B32] Field KJ, Pressel S (2018) Unity in diversity: structural and functional insights into the ancient partnerships between plants and fungi. New Phytologist 220(4): 996–1011. 10.1111/nph.15158 29696662

[B33] Field KJ, Pressel S, Duckett JG et al. (2015) Symbiotic options for the conquest of land. Trends in Ecology & Evolution 30(8): 477–486. 10.1016/j.tree.2015.05.007 26111583

[B34] Field KJ, Rimington WR, Bidartondo MI et al. (2016) Functional analysis of liverworts in dual symbiosis with *Glomeromycota* and *Mucoromycotina* fungi under a simulated Palaeozoic CO_2_ decline. ISME Journal 10(6): 1514–1516. 10.1038/ismej.2015.204 PMC5029179 26613340

[B35] Ge C, Duan Y, Han W et al. (2014) The fungal variation in rhizosphere soil of *Gentiana scabra* Bge. from Liaoning GAP base in different years. Journal of Shenyang Pharmaceutical University 31(2): 152–155. 10.14066/j.cnki.cn21-1349/r.2014.02.010

[B36] Gnanesh B, Tejaswi A, Arunakumar G et al. (2021) Molecular phylogeny, identification and pathogenicity of *Rhizopus oryzae* associated with root rot of mulberry in India. Journal of Applied Microbiology 131(1): 360–374. 10.1111/jam.14959 33277790

[B37] Go WZ, H’Ng PS, Wong M-Y et al. (2015) Occurrence and Characterisation of Mycoflora in Soil of Different Health Conditions Associated with White Root Rot Disease in Malaysian Rubber Plantation. Journal of Rubber Research 18: 159–170.

[B38] Guzmán Guzmán P, Etesami H, Santoyo G (2025) *Trichoderma*: a multifunctional agent in plant health and microbiome interactions. BMC Microbiology 25(1): 434. 10.1186/s12866-025-04158-2 PMC12255041 40652165

[B39] Hapida Y, Elfita E, Widjajanti H et al. (2021) Biodiversity and antibacterial activity of endophytic fungi isolated from jambu bol (*Syzygium malaccense*). Biodiversitas 22(12). 10.13057/biodiv/d221253

[B40] Haynes WC, Wickerham LJ, Hesseltine CW (1955) Maintenance of cultures of industrially important microorganisms. Applied Microbiology 3(6): 361–368. 10.1128/am.3.6.361-368.1955 PMC1057139 13269089

[B41] Hesseltine C, Ellis J (1961) Notes on mucorales, especially *Absidia*. Mycologia 53(4): 406–426. 10.1080/00275514.1961.12017970

[B42] Hesseltine C, Ellis J (1964) The genus *Absidia*: *Gongronella* and cylindrical-spored species of *Absidia*. Mycologia 56(4): 568–601. 10.1080/00275514.1964.12018145

[B43] Hesseltine C, Ellis J (1966) Species of *Absidia* with ovoid sporangiospores. I. Mycologia 58(5): 761–785. 10.1080/00275514.1966.12018369 5963263

[B44] Hock OG, Jeen CL, Rong CH et al. (2020) Isolation of atrazine-tolerant fungi from soil. Toxicology 16.

[B45] Hou R, Wang Y, Xu F (2019) Screening of fungal diversity and root rot antagonistic bacteria in rhizosphere soil of *Vaccinium* sp. Journal of Southwest Forestry University 39(6): 77–85. 10.11929/j.swfu.201901028

[B46] Hoysted GA, Kowal J, Jacob A et al. (2018) A mycorrhizal revolution. Current Opinion in Plant Biology 44: 1–6. 10.1016/j.pbi.2017.12.004 29289791

[B47] Ikeda I, Sugano M, Yoshida K et al. (1993) Effects of chitosan hydrolyzates on lipid absorption and on serum and liver lipid concentration in rats. Journal of Agricultural and Food Chemistry 41(3): 431–435. 10.1021/jf00027a016

[B48] Jia Z, Li C, Ma S et al. (2024) Mineral-solubilizing microbial inoculums promote *Robinia pseudoacacia* L. growth by optimizing the rhizosphere soil microbial community structure. Journal of Soil Science and Plant Nutrition 24: 6131–6144. 10.1007/s42729-024-01965-w

[B49] Junaid M, Gassing S, Muslimah A et al. et al. (2023) ‘Quantitative analysis of rhizosphere microbial population obtained from main-croplands in South Sulawesi’ IOP Conference Series: Earth and Environmental Science. IOP Publishing, 012082. 10.1088/1755-1315/1230/1/012082

[B50] Kaur R, Kaur S (2023) Carrier-Based Biofertilizers. In: Kaur S, Dwibedi V, Sahu PK et al. (Eds) Metabolomics, proteomes and gene editing approaches in biofertilizer industry. Springer Nature Singapore, Singapore, 57–75. 10.1007/978-981-99-3561-1_4

[B51] Kentjens W, Casonato S, Kaiser C (2024) Endophytic genera in californian thistle (*Cirsium arvense* (L.) Scop.). Australasian Plant Pathology 1–12. 10.1007/s13313-024-00972-w

[B52] Kim D, Gilchrist CLM, Chun J et al. (2023) UFCG: database of universal fungal core genes and pipeline for genome-wide phylogenetic analysis of fungi. Nucleic Acids Research 51(D1): D777–D784. 10.1093/nar/gkac894 PMC9825530 36271795

[B53] Komhorm A, Thongmee S, Thammakun T et al. (2021) In vivo testing of antagonistic fungi against *Alternaria brassicicola* causing Chinese kale black spot disease. Journal of Plant Diseases and Protection 128: 183–189. 10.1007/s41348-020-00382-2

[B54] Korneykova MV, Evdokimova GA (2018) Microbiota of the ground air layers in natural and industrial zones of the Kola Arctic. Journal of Environmental Science and Health, Part A 53(3): 271–277. 10.1080/10934529.2017.1397444 29125395

[B55] Kumari G, Saputhanthri P (2019) ‘Isolation and identification of heavy metal tolerant soil fungi from the Ussangoda serpentine’ Proceedings of International Forestry and Environment Symposium.

[B56] Kurniawan R (2017) Pengaruh pemberian cendawan endofit asal tanaman kelapa sawit terhadap pertumbuhan kelapa sawit pada tanah terinfeksi *Ganoderma* spp. Jurnal Agroekoteknologi Universitas Sumatera Utara 5(2): 110515.

[B57] Lee SI, Cho G, Kim SH et al. (2023) Mycobiota community and fungal species response to development stage and fire blight disease in apples. AIMS Microbiology 9(3): 554. 10.3934/microbiol.2023029 PMC10462452 37649796

[B58] Li C, Jia Z, Zhai L et al. (2021) Effects of mineral-solubilizing microorganisms on root growth, soil nutrient content, and enzyme activities in the rhizosphere soil of *Robinia pseudoacacia*. Forests 12(1): 60. 10.3390/f12010060

[B59] Li C, Wu Y, Li L et al. (2023) Different techniques reveal the difference of community structure and function of fungi from root and rhizosphere of *Salvia miltiorrhiza* Bunge. Plant Biology (Stuttgart) 25(6): 848–859. 10.1111/plb.13556 37394812

[B60] Li GJ, Hyde KD, Zhao RL et al. (2016) Fungal diversity notes 253–366: taxonomic and phylogenetic contributions to fungal taxa. Fungal Diversity 78: 1–237. 10.1007/s13225-015-0346-5

[B61] Liu T, Chen W, Zhang K et al. (2025) Three new entomopathogenic fungal species isolated from soil in China. Frontiers in Microbiology 16: 1705425. 10.3389/fmicb.2025.1705425 PMC12605131 41234740

[B62] Mandl K, Cantelmo C, Gruber E et al. (2018) Effects of glyphosate-, glufosinate-and flazasulfuron-based herbicides on soil microorganisms in a vineyard. Bulletin of Environmental Contamination and Toxicology 101(5): 562–569. 10.1007/s00128-018-2438-x PMC6223855 30229276

[B63] Manjula K, Mwangi M, Bandyopadhyay R (2005) ‘Potential of some bacteria and fungi as biocontrol agents of cassava and yam tuber rot pathogens under laboratory and green conditions’ African Crop Science Conference Proceedings, 1395–1400.

[B64] Mao Z, Zhang W, Wu C et al. (2021) Diversity and antibacterial activity of fungal endophytes from *Eucalyptus exserta*. BMC Microbiology 21(1): 155. 10.1186/s12866-021-02229-8 PMC8157698 34044780

[B65] Martin FM, Uroz S, Barker DG (2017) Ancestral alliances: Plant mutualistic symbioses with fungi and bacteria. Science 356(6340). 10.1126/science.aad4501 28546156

[B66] Martins MR, Santos C, Pereira P et al. (2017) Metalaxyl degradation by mucorales strains *Gongronella* sp. and *Rhizopus oryzae*. *Molecules* 22(12): 2225. 10.3390/molecules22122225 PMC6149714 29240696

[B67] Martins MR, Santos C, Soares C et al. (2020) *Gongronella eborensis* sp. nov., from vineyard soil of Alentejo (Portugal). International Journal of Systematic and Evolutionary Microbiology 70(5): 3475–3482. 10.1099/ijsem.0.004201 32379017

[B68] Millard PJ, Roth BL, Thi HP et al. (1997) Development of the FUN-1 family of fluorescent probes for vacuole labeling and viability testing of yeasts. Applied and Environmental Microbiology 63(7): 2897–2905. 10.1128/aem.63.7.2897-2905.1997 PMC168585 9212436

[B69] Mishra VK, Singh G, Passari AK et al. (2016) Distribution and antimicrobial potential of endophytic fungi associated with ethnomedicinal plant *Melastoma malabathricum* L. Journal of Environmental Biology 37(2): 229–237. https://doi.org/10.22438/jeb/37/02/MRN-516 27097442

[B70] Mustika TNS (2022) Potensi cendawan rhizosfer pada tegakan Jabon merah provenans Sidrap dalam melarutkan unsur hara fosfat, nitrogen, dan kalium. Universitas Hasanuddin, Makassar, Indonesia.

[B71] Nagy R, Karandashov V, Chague V et al. (2005) The characterization of novel mycorrhiza‐specific phosphate transporters from *Lycopersicon esculentum* and *Solanum tuberosum* uncovers functional redundancy in symbiotic phosphate transport in solanaceous species. The Plant Journal 42(2): 236–250. 10.1111/j.1365-313X.2005.02364.x 15807785

[B72] Nagy R, Drissner D, Amrhein N et al. (2009) Mycorrhizal phosphate uptake pathway in tomato is phosphorus‐repressible and transcriptionally regulated. New Phytologist 181(4): 950–959. 10.1111/j.1469-8137.2008.02721.x 19140941

[B73] Nwe N, Stevens WF (2002) Production of fungal chitosan by solid substrate fermentation followed by enzymatic extraction. Biotechnology Letters 24: 131–134. 10.5772/10261

[B74] Nwe N, Furuike T, Tamura H (2009) The mechanical and biological properties of chitosan scaffolds for tissue regeneration templates are significantly enhanced by chitosan from *Gongronella butleri*. Materials 2(2): 374–398. 10.3390/ma2020374

[B75] Öpik M, Moora M, Liira J et al. (2006) Composition of root‐colonizing arbuscular mycorrhizal fungal communities in different ecosystems around the globe. Journal of Ecology 94(4): 778–790. 10.1111/j.1365-2745.2006.01136.x

[B76] Partida Martinez LP, Groth I, Schmitt I et al. (2007) *Burkholderia rhizoxinica* sp. nov. and *Burkholderia endofungorum* sp. nov., bacterial endosymbionts of the plant-pathogenic fungus *Rhizopus microsporus*. International Journal of Systematic and Evolutionary Microbiology 57(Pt 11): 2583–2590. 10.1099/ijs.0.64660-0 17978222

[B77] Petrini O (1991) Fungal endophytes of tree leaves. In: Microbial Ecology of Leaves. Springer, New York, 179–197. 10.1007/978-1-4612-3168-4_9

[B78] Pici G (1955) Qualche osservazione sopra due *Mucoraceae*. Atti dell'Istituto Botanico dell'Università di Pavia 13: 38–44.

[B79] Ren Q, Khan A, Zhang J et al. (2024) Fungal community dynamics associated with the outbreaks of sugarcane root rot disease. Microbiology Spectrum 12(2): e03090-23. 10.1128/spectrum.03090-23 PMC10845956 38189328

[B80] Ribaldi M (1952) Sopra un interessante Zigomicete terricola: *Gongronella urceolifera* n. gen., n. sp. Rivista di Biologia Generale NS 44: 157–166. 14949957

[B81] Rocha I, Ma Y, Souza-Alonso P et al. (2019) Seed coating: A tool for delivering beneficial microbes to agricultural crops. Frontiers in Plant Science 10: 1357. 10.3389/fpls.2019.01357 PMC6852281 31781135

[B82] Roth R, Paszkowski U (2017) Plant carbon nourishment of arbuscular mycorrhizal fungi. Current Opinion in Plant Biology 39: 50–56. 10.1016/j.pbi.2017.05.008 28601651

[B83] Ruan YL (2014) Sucrose metabolism: gateway to diverse carbon use and sugar signaling. Annual Review of Plant Biology 65: 33–67. 10.1146/annurev-arplant-050213-040251 24579990

[B84] Santolamazza-Carbone S, Iglesias-Bernabé L, Benito-Rueda E et al. (2023) Metabarcoding of *Quercus robur* soil unveils different impact of soil microbiota on *Boletus edulis* and *B. reticulatus* mycelium concentration. https://ssrn.com/abstract=4535693 10.3390/microorganisms13092196 PMC12472445 41011527

[B85] Santos F, Garcia NFL, da Paz MF et al. (2016) Production and characterization of β-glucosidase from *Gongronella butleri* by solid-state fermentation. African Journal of Biotechnology 15(16): 633–641. 10.5897/AJB2015.15025

[B86] Seephueak P, Preecha C, Seephueak W (2019) The diversity of fungi associated with rice (*Oryza sativa* L.) from Nakhon Si Thammarat, Thailand. Thaiscience.info. https://www.thaiscience.info/Journals/Article/IJAT/10992682.pdf?utm_source=chatgpt.com

[B87] Shamshiri M, Fattahi M (2016) Effects of arbuscular mycorrhizal fungi on photosystem II activity of three pistachio rootstocks under salt stress as probed by the OJIP-test. Russian Journal of Plant Physiology 63: 101–110. 10.1134/S1021443716010155

[B88] Spatafora JW, Chang Y, Benny GL et al. (2016) A phylum-level phylogenetic classification of zygomycete fungi based on genome-scale data. Mycologia 108(5): 1028–1046. 10.3852/16-042 PMC6078412 27738200

[B89] Srivastava V (1971) Investigations into rhizosphere microflora: VIII. Light and dark treatments in relation to root-region microflora. Plant and Soil 35: 463–470. 10.1007/BF01372679

[B90] Steidinger BS, Crowther TW, Liang J et al. (2019) Climatic controls of decomposition drive the global biogeography of forest-tree symbioses. Nature 569(7756): 404–408. 10.1038/s41586-019-1128-0 31092941

[B91] Strullu Derrien C, Kenrick P, Pressel S et al. (2014) Fungal associations in *Horneophyton ligneri* from the Rhynie Chert (c. 407 million year old) closely resemble those in extant lower land plants: novel insights into ancestral plant-fungus symbioses. New Phytologist 203(3): 964–79. 10.1111/nph.12805 24750009

[B92] Sun J-Q, Huang X-X, Xu L et al. (2022) *Luteimonas saliphila* sp. nov. and *Luteimonas salinisoli* sp. nov., two novel strains isolated from saline soils. International Journal of Systematic and Evolutionary Microbiology 72(4): 005334. 10.1099/ijsem.0.005334 35471135

[B93] Tan S (1996) The chitosan yield of zygomycetes at their optimum harvesting time. Carbohydrate Polymers 30(4): 239–242. 10.1016/S0144-8617(96)00052-5

[B94] Tazi H, Hamza MA, Hallouti A et al. (2021) Biocontrol potential of nematophagous fungi against *Meloidogyne* spp. infecting tomato. Organic Agriculture 11: 63–71. 10.1007/s13165-020-00325-z

[B95] Tedersoo L, Bahram M, Põlme S et al. (2014) Fungal biogeography. Global diversity and geography of soil fungi. Science 346(6213): 1256688. 10.1126/science.1256688 25430773

[B96] Temporiti MEE, Nicola L, Girometta CE et al. (2022) The analysis of the mycobiota in plastic polluted soil reveals a reduction in metabolic ability. Journal of Fungi 8(12): 1247. 10.3390/jof8121247 PMC9785340 36547580

[B97] Teste FP, Jones MD, Dickie IA (2020) Dual‐mycorrhizal plants: their ecology and relevance. New Phytologist 225(5): 1835–1851. 10.1111/nph.16190 31514244

[B98] Thambiliyagodage C, Jayanetti M, Mendis A et al. (2023) Recent advances in chitosan-based applications-a review. Materials 16(5). 10.3390/ma16052073 PMC10004736 36903188

[B99] Thornton R (1965) Studies of fungi in pasture soils: I. Fungi associated with live roots. New Zealand Journal of Agricultural Research 8(3): 417–449. 10.1080/00288233.1965.10419888

[B100] Tibpromma S, Hyde KD, Jeewon R et al. (2017) Fungal diversity notes 491–602: taxonomic and phylogenetic contributions to fungal taxa. Fungal Diversity 83: 1–261. 10.1007/s13225-017-0378-0

[B101] Upadyay H (1969) Soil fungi from north-east and north Brizil. VII. the genus *Gongronella*. Nova Hedwigia.

[B102] Valsalan R, Mathew D, Devaki G (2022) Draft genome of *Gongronella butleri* reveals the genes contributing to its biodegradation potential. Journal of Genetic Engineering and Biotechnology 20(1): 74. 10.1186/s43141-022-00351-2 PMC9117579 35583842

[B103] Varela B, Ingrid, Durán M et al. (2017) In vitro assessment of ten strains of nematophagous fungi to control *Meloidogyne exigua*, *Meloidogyne incognita* and *Radopholus similis*. Revista Tecnologia en Marcha 30(1): 27–37. 10.18845/tm.v30i1.3062

[B104] Walder F, Niemann H, Natarajan M et al. (2012) Mycorrhizal networks: common goods of plants shared under unequal terms of trade. Plant Physiology 159(2): 789–797. 10.1104/pp.112.195727 PMC3375941 22517410

[B105] Walther G, Pawłowska J, Alastruey-Izquierdo A et al. (2013) DNA barcoding in *Mucorales*: an inventory of biodiversity. Persoonia 30: 11–47. 10.3767/003158513x665070 PMC3734965 24027345

[B106] Wang J, Zhou W, Yuan H et al. (2008) Characterization of a novel fungal chitosanase Csn2 from *Gongronella* sp. JG. Carbohydrate Research 343(15): 2583–8. 10.1016/j.carres.2008.08.004 18722595

[B107] Wang L, Tang X, Liu X et al. (2023a) Active permanent greening - a new slope greening technology based on mineral solubilizing microorganisms. Frontiers in Plant Science 14: 1219139. 10.3389/fpls.2023.1219139 PMC10498118 37711299

[B108] Wang X, Fang J, Liu P et al. (2020) *Mucoromycotina* fungi possess the ability to utilize plant sucrose as a carbon source: Evidence from *Gongronella* sp. w5. Frontiers in Microbiology 11: 591697. 10.3389/fmicb.2020.591697 PMC7874188 33584561

[B109] Wang X, Fang J, Li L et al. (2024a) *Gongronella* sp. w5 hydrolyzes plant sucrose and releases fructose to recruit phosphate-solubilizing bacteria to provide plants with phosphorus. Applied and Environmental Microbiology 90(7): e00534-24. 10.1128/aem.00534-24 PMC11267922 38904410

[B110] Wang Y-X, Zhao H, Ding Z-Y et al. (2023b) Three new species of *Gongronella* (*Cunninghamellaceae*, *Mucorales*) from soil in Hainan, China based on morphology and molecular phylogeny. Journal of Fungi 9(12). 10.3390/jof9121182 PMC10744856 38132783

[B111] Wang Y-X, Zhao H, Jiang Y et al. (2024b) Unveiling species diversity within early-diverging fungi from China III: Six new species and a new record of *Gongronella* (*Cunninghamellaceae*, *Mucoromycota*). MycoKeys 110: 287–317. 10.3897/mycokeys.110.130260 PMC11603104 39610859

[B112] Watanabe T, Moya J d D, Gonzdlez JL et al. (1996) Fungi associated with roots and fruits of black peppers in the Dominican Republic. Mycoscience 37(4): 471–475. 10.1007/BF02461007

[B113] Wei Y-H, Liou G-Y, Tzean S-S et al. (2024) *Gongronella fusoacuminata* (*Cunninghamellaceae*, *Mucorales*), a new species from Taiwan. Phytotaxa 678(3): 185–192. 10.11646/phytotaxa.678.3.4

[B114] Wipf D, Krajinski F, van Tuinen D et al. (2019) Trading on the arbuscular mycorrhiza market: from arbuscules to common mycorrhizal networks. New Phytologist 223(3): 1127–1142. 10.1111/nph.15775 30843207

[B115] Wu C, Yang Y, Wang Y et al. (2024) Colonization of root endophytic fungus *Serendipita indica* improves drought tolerance of *Pinus taeda* seedlings by regulating metabolome and proteome. Frontiers in Microbiology 15: 1294833. 10.3389/fmicb.2024.1294833 PMC10978793 38559354

[B116] Wu S, Gao Z, Bai M et al. (2023) Response relationship between different incidence degrees of tobacco black shank disease and rhizosphere microorganisms. Soil Fertility Science China (7): 223–231.

[B117] Wu Y, Zhang J, Guo X et al. (2017) Isolation and characterisation of a rock solubilising fungus for application in mine-spoil reclamation. European Journal of Soil Biology 81: 76–82. 10.1016/j.ejsobi.2017.06.011

[B118] You Y-H, Park JM, Seo YG et al. (2017) Distribution, characterization, and diversity of the endophytic fungal communities on Korean seacoasts showing contrasting geographic conditions. Mycobiology 45(3): 150–159. 10.5941/MYCO.2017.45.3.150 PMC5673510 29138619

[B119] You YH, Sung HJ, Nguyen MH et al. (2023) Unrecorded fungi isolated from rhizosphere soil of *Fallopia sachalinensis* in Dokdo Islands. Korean Journal of Mycology 51(3): 251–257. 10.4489/kjm.20230027

[B120] Yuan G, Jiang F, Sun Y et al. (2021) Isolation, purification and ITS molecular identification of pathogenic fungi of strawberry root rot disease. Modern Agricultural Science and Technology (05): 103–107.

[B121] Zakeel MC, Geering AD, Akinsanmi OA (2023) A fungal pathogen is unlikely to be the cause of abnormal vertical growth syndrome in macadamia. Plant Pathology 72(4): 731–741. 10.1111/ppa.13689

[B122] Zhang H, Yang Z, Jiang Z et al. (2023) Diversity of fungi isolated from potato nematode cysts in Guizhou Province, China. Journal of Fungi 9(2): 247. 10.3390/jof9020247 PMC9965506 36836361

[B123] Zhang Q, Dong C, Liang Z et al. (2021) Community composition and diversity of culturable endophytic fungi in bark of *Eucommia ulmoides* from different regions of China. Mycosystema 40(10). 10.13346/j.mycosystema.210145

[B124] Zhang X, Gao G, Wu Z et al. (2019a) Agroforestry alters the rhizosphere soil bacterial and fungal communities of moso bamboo plantations in subtropical China. Applied Soil Ecology 143: 192–200. 10.1016/j.apsoil.2019.07.019

[B125] Zhang Z-Y, Han Y-F, Chen W-H et al. (2019b) *Gongronella sichuanensis* (*Cunninghamellaceae*, *Mucorales*), a new species isolated from soil in China. Phytotaxa 416(2): 167–174–167–174. 10.11646/phytotaxa.416.2.4

[B126] Zhao H, Nie Y, Zong T-K et al. (2023) Species diversity, updated classification and divergence times of the phylum *Mucoromycota*. Fungal Diversity 123(1): 49–157. 10.1007/s13225-023-00525-4

[B127] Zhen Q, Wang X, Cheng X et al. (2024) Remediation of toxic metal and metalloid pollution with plant symbiotic fungi. Advances in Applied Microbiology 129: 171–187. 10.1016/bs.aambs.2024.04.001 39389705

[B128] Zhou W, Yuan H, Wang J et al. (2008) Production, purification and characterization of chitosanase produced by *Gongronella* sp. JG. Letters in Applied Microbiology 46(1): 49–54. 10.1111/j.1472-765X.2007.02262.x 17983432

[B129] Zuraina MY, Abdul Khalil K, Qursyna N et al. (2022) Molecular identification of fungi associated with plant rot disease of soursop (*Annona muricata*) in Seri Menanti, Negeri Sembilan. Malaysian Journal of Biochemistry and Molecular Biology 25(3): 18–26.

